# Dimension Effects on the Acoustic Behavior of TRC Plates

**DOI:** 10.3390/ma13040955

**Published:** 2020-02-20

**Authors:** Nicolas Ospitia, Dimitrios G. Aggelis, Eleni Tsangouri

**Affiliations:** Department of Mechanics of Materials and Constructions (MeMC), Vrije Universiteit Brussel, 1050 Brussel, Belgium; Nicolas.Ospitia.Patino@vub.be (N.O.); Eleni.Tsangouri@vub.be (E.T.)

**Keywords:** acoustic emission, wave propagation, wave analysis, textile-reinforced cementitious (TRC) composites, bending

## Abstract

Acoustic emission (AE) is a monitoring technique that has proven its suitability in and outside of the laboratory in characterizing the structural condition of materials. In composites for construction and repair, several breakthroughs have been recently noted involving mainly fracture mode evaluation based on the AE waveform characteristics. However, the acquired signals, apart from the cracking source strongly depend on the size and shape of the plate specimens. While the effect of wave propagation distance has been studied, the effect of the lateral dimension of the plate has not been given proper attention, being a broken link in translating the results from small coupons to real size plates. This paper examines wave propagation from artificial sources as well as actual AE signals in textile-reinforced cement (TRC) plates indicating the strong differences in the results that are attributed just to the shape and size of the specimens and showing that interpretation toward the actual sources is firmly connected to geometric factors.

## 1. Introduction

**Figure 1:**
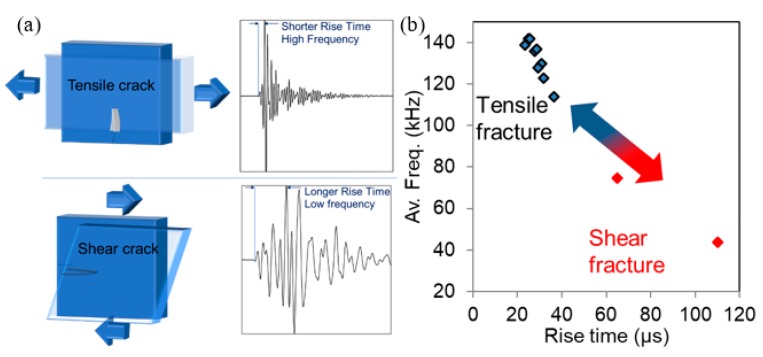
(**a**) Schematic representation of fracture modes and corresponding acoustic emission (AE) waveforms; (**b**) Av. Freq. vs. rise time plot from bending of textile-reinforced cement (TRC) beams taken from [18].

Acoustic emission (AE) monitoring is a very promising method for the evaluation of the condition of an existing structure. The advancements in electronics and the relatively easy application, combined with the sensitivity of the technique render it one of the most encouraging technologies for damage assessment. In the field of structural materials monitoring, elastic waves recorded after fracture propagation events are used among others for damage localization [1,2,3], evaluation of structural integrity based on specific indices [4,5,6], corrosion monitoring [7,8], as well as characterization of the fracture mode [9,10,11,12]. It has been well demonstrated that tensile and shear or mixed mode cracks can be differentiated by the wave frequency content and the duration (or other waveform parameters) [13,14,15], see also Figure 1a,b. In the field of composites for construction, which are the continuously advancing, more sustainable, lightweight alternative of concrete, and specifically for thin plate TRCs (textile-reinforced cements), acoustic monitoring is imperative, since the TRC is not transparent making visual inspection of the plate itself and the underlying substrate impossible. AE has recently shown the ability to characterize the fracture mode (cracking, debonding of successive textile layers from the cement or detachment from the concrete substrate) [16,17]. Furthermore, AE proved sensitive to the type of strains that will eventually lead to a specific damage process, allowing the prediction of the dominant fracture mode [18,19]. Therefore, in laboratory settings and mainly beam geometries, AE has demonstrated high capacity in characterization, which is extremely useful in material development studies. Fracture mode classification is attempted mainly based on two waveform parameters, average frequency (AF) and RA or rise time (RT). AF is the number of threshold crossings over the waveform duration (DUR), RT is the delay between the first threshold crossing and the peak amplitude, A, and RA is the ratio between RT and the amplitude. According to the recommendations [6], the low frequency and higher RA or RT represent the mixed or shear mode events, while higher frequency and shorter signals (lower RT) represent tensile cracking (see Figure 1b). It is always highlighted that the importance is in the relative difference or shift between the populations, not in the absolute separation that always depends on the experimental conditions, like sensor’s sensitivity, propagation distance, material properties, etc. This shift is present during the fracture evolution in a TRC beam, as has been demonstrated several times recently [16,17,18,19]. Practically, when the mode shifts from cement cracking to delaminations, the frequency decreases by approximately 50% and RT increases by an even more significant proportion [18]. 

However, in larger scale, several shortcomings do not allow the accurate interpretation of AE results allowing only the utilization of the basic features, like the cumulative AE numbers and source localization. Frequency content and waveform shape are severely distorted especially in heterogeneous media because of scattering, damping, reflections, as well as wave dispersion (when dealing with plate geometries) [20,21]. In that case separation between the “symmetric” and “antisymmetric” wave modes occurs as the first (S0) propagates with higher speed than the latter (A0). Therefore, the shift illustrated in Figure 1b, may be a result of the propagation conditions that mask the characteristics of the source. Indeed, the propagation distance has been shown to be a crucial factor influencing the classification [14,22,23] because of the more effective attenuation of the higher frequencies as well as the “spreading” of the waveforms in the time domain. Apart from experimental AE studies, there are several numerical works on the subject of wave propagation in plates, some examples are shown in [24,25,26]. The effect in the final signal acquisition, as acknowledged in these studies, is quite severe masking the initial characteristics of the AE source. In addition, a secondary effect, especially in plates, where the wave propagation is parallel to the plate and the sensor contact plane, comes from aperture effects or the interaction between the wavelength and the sensor’s diameter that starts to become important [27,28,29,30], since the concept of point receiver is no longer valid. Therefore, in general, the upgrade from laboratory coupons to realistic geometries needs further investigation as the same classification between AE signals from different fracture modes, as examined in small coupons, may not apply for larger scale. 

However, it is not only the wave propagation distance relevant to the final application. Reinforcement can be applied in the form of narrow strips (typically carbon fibre composites, CFRP) as well as wider plates (TRC) [17]. Therefore, the effect of the vertical dimension (width) of the plates is also important as highlighted in the past [31,32] for homogeneous specimens and evaluated for the first time for TRC materials herein. The present paper aims to examine the influence of the different dimensions (width and thickness) of the plate on the AE signals. When the width of the plate is small, the geometry is closer to a beam and the wave propagation is essentially one-dimensional leading to strong waveguide behavior without allowing spreading of the beam. For larger widths though, the propagation travels in a circular wave front, the energy is spread to a larger area of the plate, and wave travels longer until it is possibly reflected at the edges. 

In order to study the effect of the geometry in a controlled way, first wave measurements were conducted in plate and beam geometries using artificial AE (pencil lead excitation). Despite that the excitations are applied on the external surface of the sample and are not “buried” as would be an actual internal crack, they offer a means of studying the general phenomenon of propagation and the influence of the geometry. Then, mechanical bending tests of the same specimens took place with concurrent AE monitoring. In this case the excitation was given by the actual fracture events from the start of loading until the failure of the composite. Results show that considerable differences arise between beams and plates, not allowing generalization of the outcome from laboratory specimens to realistic sizes without proper attention.

## 2. Experimental Details

### 2.1. Materials and Loading

**Figure 2:**
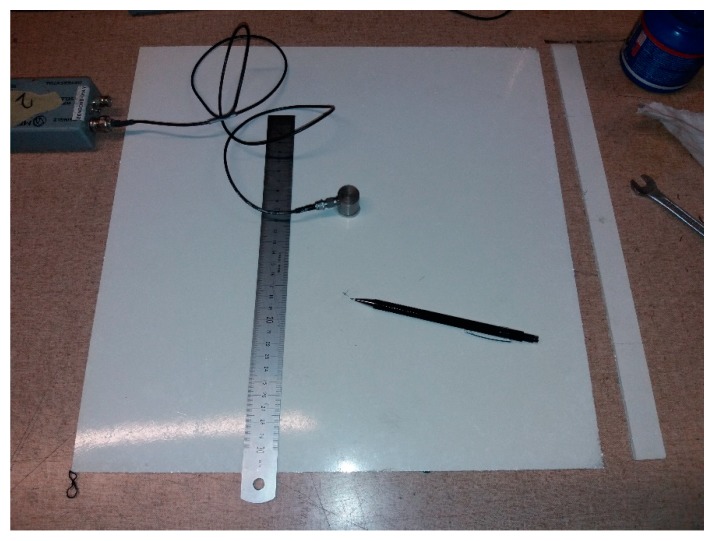
The TRC plate (400 × 400 mm^2^, **left**) and beam (400 × 20 mm^2^, **right**).

Similar TRC specimens have been studied recently and their manufacturing is shown in [17,18]. For completeness, the basic details are also reported here. The matrix consists of inorganic calcium phosphate cement (IPC), which is a mixture of a calcium silicate powder and a phosphoric acid-based solution of metal oxides. The weight ratio of powder to liquid is 0.8. For the mixing a Heidolph RZR 2102 overhead mixer (Analis, Suarlee, Belgium ) was used. First, the liquid and the powder were mixed at 250 rpm until the powder was merged into the fluid, after which the speed was increased to 2000 rpm. E-glass chopped fiber mats with a surface density of 300 g/m^2^ (Owens Corning M705- 300, Owens Corning, Brussels, Belgium ) were used as reinforcement. TRC laminates were made by hand layup with an average matrix consumption of 800 g/m^2^ for each layer, which results in an average fiber volume fraction (V_f_) of 20%. The laminates were cured in ambient conditions for 24 h. Post-curing was performed at 60 °C for 24 h while both bottom and top planes were covered with a plastic film to prevent early liquid evaporation. Plate and beam samples were cutoff from the different laminates. The dimensions of the TRC plate were 400 × 400 × 2.5 mm^3^ and for the beam they were 400 × 20 × 4.5 mm^3^. A photograph of the specimens is shown in Figure 2.

### 2.2. Wave Propagation Experiments

**Figure 3:**
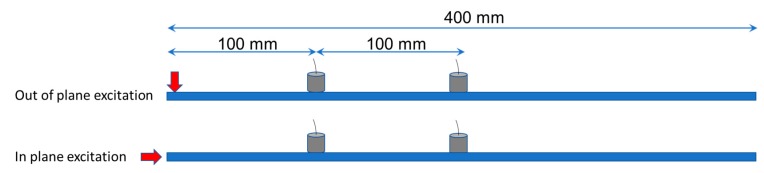
Schematic representation of the AE measurements with pencil lead break excitation.

For the pencil lead break measurements, two AE sensors, resonant at 150 kHz, were placed with a separation distance of 100 mm, while the first was placed another 100 mm from the edge of the plate or beam (see Figure 3). Excitations by the pencil lead (HB 0.5, length approximately 4 mm) took place both at the top plane, creating thus an out-of-plane excitation (top of Figure 3) as well as at the vertical side of the specimen to produce in-plane motion (bottom of Figure 3). Because of the orientation of the excitation, the first case can be considered close to an axial defect or a delamination in the TRC plate or beam, while the latter is closer to a matrix crack that may open due to tension. The waveform analysis focuses on the response of the furthest sensors at 200 mm from the excitation, while the first sensor was used for experimental reference and triggering purposes. Propagation speed, as measured by the onset of the waveforms between the first and last receiver was 2730 m/s. 

### 2.3. AE Monitoring During Mechanical Test

**Figure 4:**
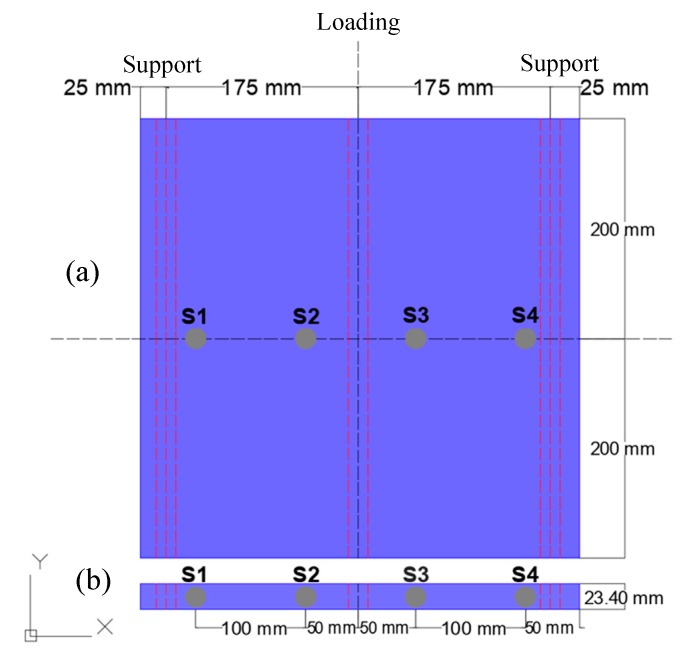
Schematic representation of three-point bending loading points and supports, and sensors distribution for both: (**a**) TRC plate and (**b**) TRC beam. S1 to S4 stand for AE sensors.

**Figure 5:**
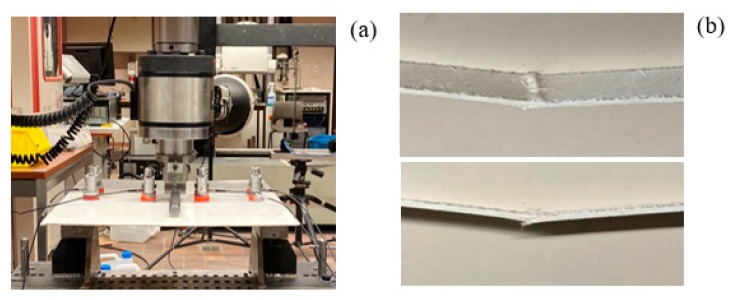
(**a**) Three-point bending setup for the TRC plate (the same setup was used for the TRC beam). The magnetic clamps holding the four AE sensors are visible on the plate. (**b**) Fractured TRC beam after the end of the test.

The TRC samples were loaded in a three-point bending test. The span between the supports was set to 350 mm, see Figure 4. The test was performed using an Instron 5885H universal testing machine (Instron, Île-de-France, France ) using a loading rate of 2 mm/min. Figure 5a,b show photographs of the setup and two close ups of the fracture area after the end of the test, respectively. AE monitoring was performed with four AE sensors (similar to the ones used in Section 2.2) with a resonant frequency of 150 kHz. The sensors were placed at 50 mm and 150 mm from the center, on both sides, for both samples, as shown in Figure 4. Vaseline was used as a coupling between the sensor and the samples. Signals greater than 50 dB (threshold) were pre-amplified by 40 dB and recorded by a Micro-II Digital AE System (Mistras Group, Princeton, NJ, USA) with a sampling rate of 5 MHz. The AE activity with their corresponding waveforms were recorded and analyzed in this manuscript. The focus was given in how the geometry of the sample influences the different AE parameters and waveforms generated from similar loading conditions, with the same nominal propagation distance. 

## 3. Results from Artificial Excitation

**Figure 6:**
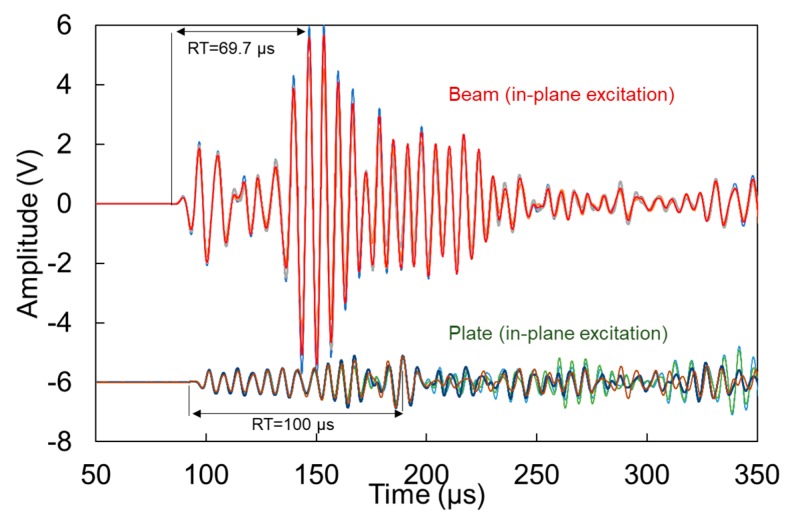
Waveforms received after pencil lead excitation on the side of the specimens (in-plane excitation) for beam and plate geometries.

Figure 6 shows typical waveforms after pencil lead break on the side of the beam (or plate) simulating a matrix crack (in-plane excitation), as received by the furthest (2nd) sensor. Waveforms from four individual excitations are included to show the repeatability of the test. The energy is quite strong as evidenced by the relatively large amplitudes. Specifically, and despite the 200 mm long propagation, the amplitude on the beam reaches 6 V, corresponding to 95 dB in the system. For reference, it can be said that the 1st sensor at 100 mm recorded a saturated waveform at 10 V (not presented), which is the limit of the acquisition board. In the response of the 2nd sensor shown on top of Figure 6, a weak separation of modes is formed, with the first burst at 100 μs and the second peaking at 150 μs. The corresponding sensor on the plate geometry shows a much lower amplitude (bottom of Figure 6) because of the spreading of the wave front which is essentially restrained in the beam geometry. Additionally, its peak comes clearly later reaching a RT of 100 μs, longer compared to the RT of the corresponding signal on the beam (69.7 μs).

**Figure 7:**
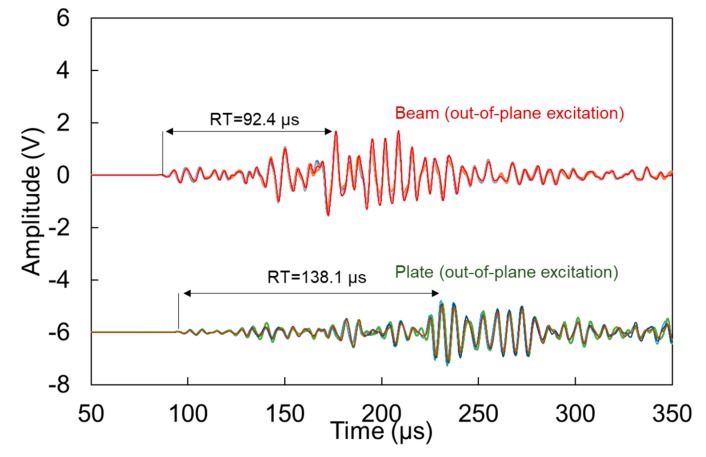
Waveforms received after pencil lead excitation on the surface of the specimens for beam and plate geometries.

The waveforms for the out-of-plane excitation, are shown in Figure 7. This excitation implies that the dominant mode is the “antisymmetric” A0, which is arguably slower than the symmetric (S0) [10], as will also be demonstrated below. Therefore, reasonably the main content of the waveforms arrives later, resulting in longer RT values than the corresponding cases of Figure 6. Again, as the wave in plates spreads in two dimensions, it is reasonable to expect lower amplitude for the plate than the beam. In addition, the strongest burst of energy arrives later in the plate geometry than the beam. This influences the RT of the waveforms with the plate RT being 40% longer, while the amplitude is approximately half. This is attributed to the aforementioned spreading of the energy on the plate geometry, as well as the possible absence of reflections from the edges, that are bound to influence the beam in a stronger way. 

**Figure 8:**
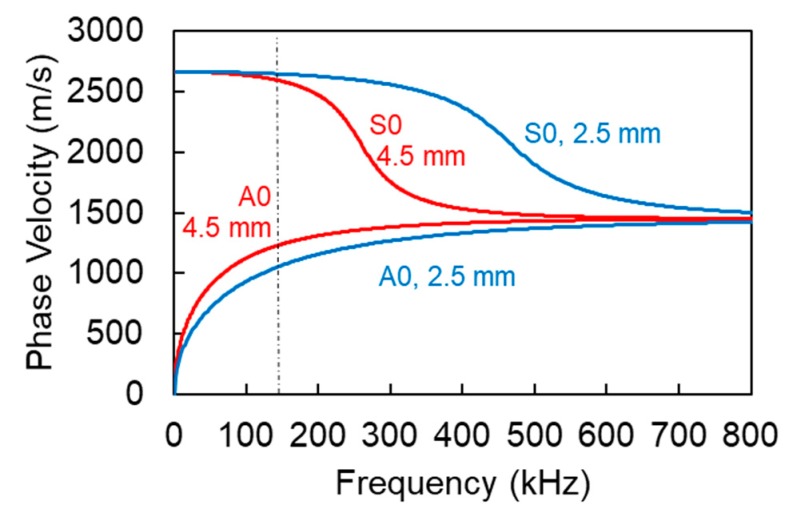
Dispersion curves for S0 and A0 modes for TRC with different thickness and longitudinal and shear wave velocity 3000 m/s and 1550 m/s respectively. The vertical dash-dot line denotes the resonant frequency of the receivers.

Figure 8 demonstrates the calculated indicative dispersion curves for both thicknesses and material with longitudinal and shear wave velocities 3000 m/s and 1550 m/s respectively. The results show that for the frequency of interest (sensors resonant at 150 kHz) the S0 mode for both cases is expected much faster than the A0, with phase velocities of approximately 2600 m/s and 1100 m/s respectively. This is also in accordance with the experimental observation that most of the content of the waveform from in-plane excitation arrives earlier than the out-of-plane, which can be seen by the shorter RT values in Figure 6 than the RT values of Figure 7. Differences related to the thickness are much smaller and specifically for the S0 the difference is less than 40 m/s and thus may not have a certain effect on the onset of the waveforms. The difference is a little higher for the A0 mode, where the thick laminate exhibits 170 m/s higher velocity than the thin one, something that could contribute to the shorter RT exhibited by the beams compared to the plates and seen in Figure 6 and Figure 7. It should be highlighted that these dispersion curves are theoretical and that in practice both wave modes are present; therefore, strong differences in the onset of the waveforms are not seen because of the S0 arrival in both cases with more or less the same velocity. Differences are more pronounced in the amplitude axis, where the in-place excitation results in much higher S0 burst than the out-of-plane one. 

Table 1 summarizes some basic descriptors of the waveforms. Comparisons can be made between beam and plate geometries, as well as in- and out-of-plane excitations. For both types of excitation, the plate consistently exhibits longer RT by about 40 to 50% than the beam. In addition, it exhibits lower amplitude(Amp) by several dB, a difference that is more pronounced in the in-plane excitation. Frequency indicators are discussed in more detail in the next section along with Fast Fourier Transformation (FFT) analyses of the waveforms but in any case, the peak frequency (or the frequency with the higher magnitude, PF) is slightly higher for the beam.

Moving to the comparison between the different excitations for the same geometry, the in-plane one (simulating vertical crack) shows shorter RT, something reasonable as this orientation of excitation would excite mostly the S0 mode which is faster compared to the A0 excited by a delamination. This is also validated by several AE studies, characterizing cracks and delaminations on composites [33,34], and also mentioned in studies on TRCs [16,18].

The differences in the waveform shape and parameters are quite strong and would definitely influence any characterization approach. It is evident that a typical waveform after a matrix crack on a beam has little similarity to a corresponding waveform in a plate, something obvious even by visually comparing the top and bottom waveforms of Figure 6 and Figure 7.

It is interesting to discuss these results in the light of an older numerical study, which interprets the higher amplitude on an aluminum beam as an effect of the immediate reflections, which are not present in the case of a plate of the same material [31]. These reflections, according to [31], are responsible for the higher amplitude, duration, and energy for the narrow coupon relatively to the larger field-sized sample. In addition, it is also mentioned in this study that the frequency is “distorted” in the narrow sample because of the edge reflections, without however explicitly discussing a shift to higher or lower frequencies for one or the other case. The lower amplitude detected in plates may reduce the possibility to capture a fracture signal in a plate, or underestimate the importance of a weak signal, while in a beam, having this “magnification” effect due to the reflections, signals are more easily recorded. This interpretation is perfectly compatible with the one made above about the differences between higher energy in the beam because of the 1D propagation (waveguide effect in beam) and 2D propagation (plate) with more spreading and thus lower energy content. On the other hand, as also mentioned in [30], the multiple reflections in beams, distorts the signal, possibly showing lack of consistency because of the multiple interferences.

**Figure 9:**
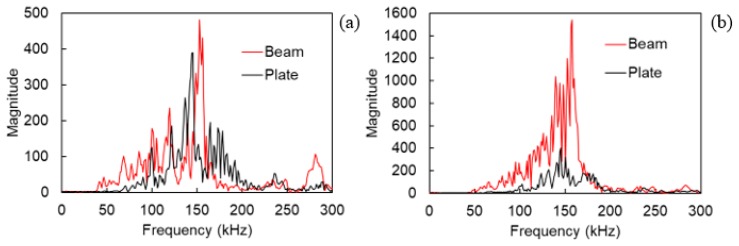
FFT of waveforms received 200 mm away from a pencil lead excitation (**a**) on the surface (out-of-plane), and (**b**) on the side (in-plane), of a beam and a plate geometry.

Concerning the frequency content in the present study, a dramatic change in the bandwidth between beams and plates was not observed (Figure 9). The waveform of the beam exhibits obviously higher magnitude for both excitations, something consistent with its higher time domain amplitude, but the content lies in the same band as in the plate (essentially 50 to 200 kHz). Still one difference can be seen, concerning the maximum peak (peak frequency, PF), which for the beam is in both cases higher than 150 kHz while for the plate is just below that value, as also seen in Table 1. In any case, the specific sensors are resonant at 150 kHz and therefore, any difference is reasonably compressed around this value. 

## 4. AE Results and Comparison

The same specimens were mechanically tested in order to assess their AE behavior, when the excitation comes from the actual cracking of the material and not from a simulated source. Apart from the width of the specimens, the three-point-bending experimental setup remained identical between the two specimens to allow comparisons and the evaluation of the width dimension influence. 

**Figure 10:**
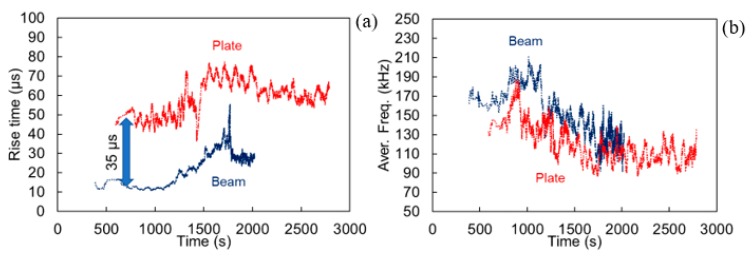
(**a**) Rise time and (**b**) average frequency vs. time of test. The lines are sliding averages of 200 successive data points.

Since the focus of the paper is not the load-bearing capacity of the materials, load histories are not presented for brevity. Figure 10a demonstrates the RT as received by the sensor S2 (50 mm from the center) for both the plate and the beam specimen. The data are presented in terms of their sliding average of 200 points. The sliding average is a suitable way to represent AE data in order to show the general trend, compressing the high experimental scatter which is inherent to fracture and therefore, the AE data [6,35]. For the beam, RT early values stand at approximately 15 μs, while the plate exhibits a much higher average at approximately 50 μs. These data recorded at the beginning of the test and at low load levels can be safely attributed to matrix cracking in both cases, showing how differently the same type of source is recorded in a beam and a plate specimen. Later, as is normal for this material, RT increases symbolizing the gradual shift to shear events like pull-out and delaminations. Concerning the frequency characteristics, still a difference can be observed in Figure 10b, but it is much milder, something expected because of the resonant behavior of the sensors. Matrix cracking is exhibited with an average value of 170 kHz on the beam and with approximately 140 kHz on the plate.

**Figure 11:**
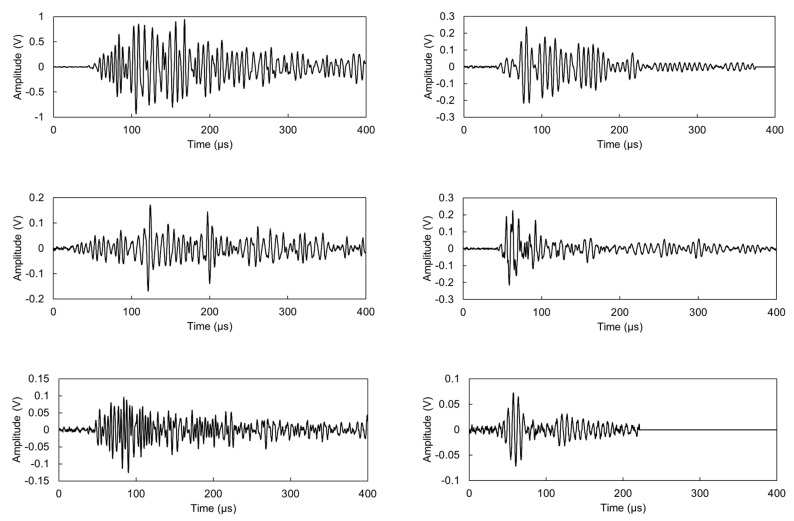

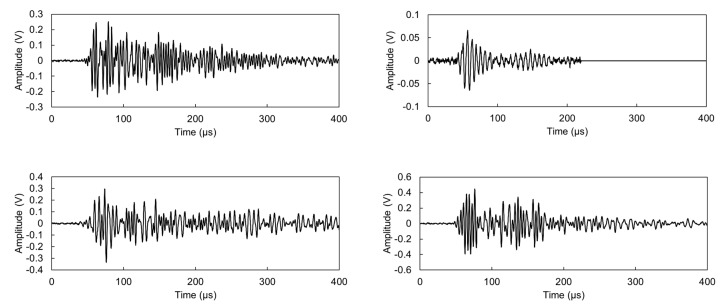
AE signals attributed to cracking from plate (**left**) and beam (**right**).

**Figure 12:**
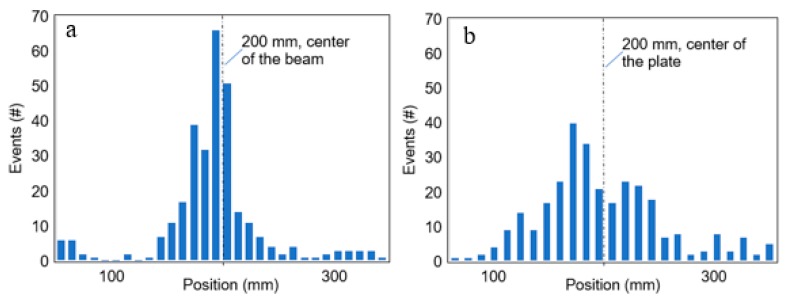
Linear AE localization distribution for the first 300 events of the (**a**) beam and (**b**) plate specimens.

During the test several thousands of AE waveforms were recorded. Selecting and presenting “representative” ones is a challenge but it is always important to manifest the raw information, from which the parameters are calculated. Therefore, five waveforms were selected from the early loading stage of the beam and the plate (within the first 300 recorded events), attributed, therefore, to matrix cracking. They were chosen based on the similarity of their parameters to the average of the class, as shown in Table 2. These waveforms are depicted in Figure 11, while Figure 12 shows the localization results for these 300 first events, confirming that the sources mostly stand at the middle zone of the span, where the bending moment is maximum and the material more prone to cracking.

As seen in the examples of Figure 11 as well as the average results of Table 2, most of the trends seen in the earlier section using pencil lead excitation are confirmed. In general, waveforms attributed to matrix cracking are shorter in beams as indicated by the lower RT and duration average values. In addition, they generally possess higher frequency characteristics, as measured by the AF equal to 170 kHz for beams and 143 kHz for plates. The values of initiation frequency (IF, essentially the average frequency of the first part of the waveform until the peak amplitude) show even stronger changes between the two geometries, specifically, 389 kHz for beam and 274 kHz for plate. In general, although usually, AF is used in analyses, it is shown that IF has very strong characterization power as it is calculated by the initial part of the signal, which is less influenced by reverberations of the piezo element, similarly like RT is more usually examined than the whole duration of the waveforms. Since, as aforementioned, shear events are escorted by higher RT, the higher values of RT for the plate compared to the beam could be wrongly interpreted as a manifestation of shear signals while the actual origin is just the difference in the geometry and wave propagation conditions.

Signals from shear are also present in the dataset at later times. However, they are normally overlapping with the cracking ones and therefore, the analysis herein considers only the early part of the data set (first 300 hits) that can safely be attributed to a single fracture type (cracking), while the load was up to 40% of the maximum. 

The analysis of the energy-related parameters such as amplitude (dB) or absolute energy (attoJ) deserve a special mention. It is clear from Table 2 that the plate geometry, exhibits much higher amplitude, posing the only discrepancy between the simulated pencil lead break tests and the actual AE tests. This may initially seem contradictive to the geometric attenuation effect, since it is known that propagation in the plate exhibits stronger spreading of the wave front, as already discussed above. Nevertheless, one should not neglect that in the previously mentioned artificial excitation measurements, the excitation came from exactly the same type and size of the source (pencil lead) and at the same distance. In the actual test however, the length and width during any crack propagation increment cannot be directly controlled. While in a beam the crack length is limited by the beam’s width, there is no such limitation for the case of plate, something that contributes to a stronger release of energy. Therefore, although geometric and recording parameters can be controlled in model tests being identical to a mechanical test, the actual excitation energy may still pose a difference.

The main aim of the paper is to demonstrate that there are significant discrepancies between AE monitoring behaviors depending on the shape of the elements. This is relevant because most of the laboratory tests take place in small coupons, whereas the actual application of the material is in the form of large plates. The deviations are quite large since for example, as shown in Table 2, the duration of a typical cracking signal in plate (realistic engineering shape) is approximately eight times longer than in a laboratory specimen (beam). Relying only on the results of a laboratory sized sample, the cracking AE waveforms in a large plate would be certainly misinterpreted as delaminations because of their much longer duration, RT, and other characteristics. 

The results will have application on TRC skins that are used either as strengthening/repair additional layers or as standalone load bearing members (i.e., façade sandwich panels). Although TRC materials have significant development in their design and mechanical response optimization, the characterization of the fracture behavior is much more complicated than other relatively homogeneous plate elements. Therefore, studies on the fracture and AE behavior of TRCs increase the confidence in the use of this lightweight and thus more sustainable material than traditional concrete.

## 5. Conclusions

The behavior of elastic waves in plates is crucial for the interpretation of AE signals. AE as usually monitored in beam geometries in laboratory exhibits strong discrepancies from plate geometries, commonly found in real life applications of composite materials. The main conclusions can be summarized as follows:

- In-plane excitation on a TRC sample (simulating matrix crack) produces shorter AE waveforms than the corresponding out-of-plane excitation, clearly showing that fracture mode characterization based on AE is possible in the cementitious composites.
- Plate geometries exhibit longer waveform characteristics like RT and duration, and slightly lower frequency for the same artificial excitation (pencil lead break).
- Cracking signals from actual mechanical testing show the same trends with artificial excitation between beams and plates, with a difference on the energy-related parameters, that seem higher for plates. This is attributed to the unrestricted crack dimensions and propagation increments in the large geometries.

The differences on AE waveform descriptors reported herein show that proper care should always be taken if the results from laboratory coupons tests are to be expanded in realistic components inspection. Additionally, the thickness of the plate is a parameter that should be taken into consideration. In any case, the enlargement of laboratory coupons to resemble plates instead of beams is desirable.

## Figures and Tables

**Table 1:** Basic waveform descriptors under different excitation in beam and plate geometry.

	Out of Plane (Simulating Delamination)	In Plane (Simulating Cracking)	Out of Plane (Simulating Delamination)	In Plane (Simulating Cracking)	Out of plane (Simulating Delamination)	In plane (Simulating Cracking)
	RT (μs)	RT (μs)	Amp (dB)	Amp (dB)	PF (kHz)	PF (kHz)
beam	92.4	69.7	84.6	95.1	152.6	157.5
plate	138. 1	100	80.0	78.8	145.3	145.3

**Table 2:** Basic waveform descriptors of cracking signals in beam and plate geometries.

	RT (μs)	DUR (μs)	Amp (dB)	AF (kHz)	IF (kHz)
beam	14.0	65.6	56.4	170.1	389.1
plate	46.0	335.3	60.4	136.6	274.3
